# Including Quantum Effects in Molecular Simulations Using the Feynman–Kleinert Linearized Path Integral Method

**DOI:** 10.3390/e27070702

**Published:** 2025-06-30

**Authors:** Jens Aage Poulsen, Gunnar Nyman

**Affiliations:** Department of Chemistry and Molecular Biology, University of Gothenburg, SE 413 90 Gothenburg, Sweden; jens.poulsen@gu.se

**Keywords:** Wigner distribution, classical trajectories, Feynman path integral, Boltzmann distribution

## Abstract

During the last few decades, several approximate, but useful, methods for including dynamical quantum effects in molecular simulations have been developed. These methods can be applied to systems with hundreds of degrees of freedom and with arbitrary potentials. Among these methods, we find the Feynman–Kleinert linearized path integral model, including its planetary versions, which are the focus of this review. The aim is to calculate quantum correlation functions for complex systems. Many important properties, e.g., transport coefficients, may thus be obtained. We summarize important applications of the method, and compare them to alternative ones, such as centroid molecular dynamics and ring polymer molecular dynamics. We finally discuss possible future improvements of the FK-LPI method.

## 1. Introduction

Molecular dynamics (MD) simulations have become an increasingly important tool for studying atomic and molecular proceses in chemistry, physics and biology [1,2,3,4,5]. There are several reasons for this. Firstly, the associated force-fields have become more elaborate and now even allow for bond-breaking, as is exemplified by the so-called Reax-FF model [6]. Even Density Functional Theory (DFT) methods can be adopted for computing the forces in MD simulations, and many DFT MD packages are available; see Ref. [7] as an example. Secondly, the increase in computer power trivially favors numerical methods such as MD, and with the availability of deep learning methods, AI-generated potential energy surfaces and force-fields may further reduce the computer time of MD simulations [8].

Traditional MD routines are based on integrating classical equations of motion, like Newton’s or Hamilton’s equations of motion, and the relative simplicity of these allows for MD to tackle very large systems. However, these equations have a limited range of applicability. Indeed, there are two scenarios where they may be invalid. The first case is when dealing with light nuclei, when quantum effects such as tunneling and vibrational zero-point motion can be significant even at room temperature. The second case is when studying systems at low temperature, which also makes quantum effects more important. Generally speaking, quantum effects are important when the typical vibrational energy spacings of the system are larger than the accessible thermal energy: $\hbar \omega /k_{B}T\gg 1$. Well-known examples are reactions involving hydrogen atoms and low-temperature liquids.

In the last few decades, extensions of Newton’s equations have been implemented which are able to approximately describe dynamical quantum effects while still simulating hundreds of atoms interacting through realistic potentials. Perhaps among the most notable of these are ring polymer molecular dynamics (RPMD) [9,10], centroid molecular dynamics (CMD) [11,12,13] and the classical Wigner [14,15,16,17] models. These methods represent extensions of traditional MD schemes and they can be combined with any subroutine that supplies the gradient of the system’s potential energy. There are of course other computational models that simulate quantum effects in complex systems, for instance pure path integral methods, but these typically limit the bath potential energy function to quadratic potentials only. For a recent extensive review of such methods, we refer to Ref. [18].

RPMD, CMD and the classical Wigner methods target the calculation of so-called quantum correlation functions of the system. Once obtained, these provide extensive information on the system dynamics. The three methods all have their origin in statistical mechanics through Feynman path integrals and are not based on wavefunctions. A short description of each method is provided below.

RPMD replaces each atom in a simulation by a Feynman ring polymer necklace that contains multiple copies of the same physical atom, coupled together by harmonic spring terms, thereby giving rise to positional quantum effects. The copies in the necklace are refered to as “beads”. The classical-like Hamiltonian which describes this model is adopted from the imaginary time path integral of the Boltzmann operator and it is a postulate of RPMD that dynamical information can be obtained by performing classical MD in this extended phase space containing the necklace [9,10]. Generally, within RPMD, the average value over all beads of the estimator is used in the evaluation of the correlation function.

CMD is closely related to RPMD. The central difference between them is that in CMD, the higher-order Fourier modes of the necklace are integrated out, leaving only the centroid as a dynamical variable. As a consequence, the latter moves on an effective potential, refered to as the centroid potential [10,11].

The classical Wigner (CW) model introduces quantum effects into molecular simulations by sampling initial phase-space points from a quantum Wigner distribution of the Boltzmann operator. The phase-space points are then propagated classically using Newton’s laws of motion. Thus, the CW model may be seen as a hybrid method using quantum initial conditions and classical trajectories for the time evolution. The Feynman–Kleinert linearized path integral (FK-LPI) model is a special implementation of the CW model.

The FK-LPI model, which is the subject of this review, has been used successfully in a number of condensed phase problems such as diffusion in low-temperature para-hydrogen [19] and helium [20], establishing a quantum model of liquid water [21] and computing the spectra of density fluctuations for low-temperature liquids [22,23,24,25,26,27]. FK-LPI and other classical Wigner implementations do not preserve the quantum canonical ensemble during the classical MD. In practice this means that vibrational zero-point energy may leak into intermolecular modes and thereby heat up the liquid [28]; see more below. Perhaps this is the main flaw of the classical Wigner method, seen both from a conceptual and a computational point of view. After its inception, FK-LPI has also been formulated in a special “planetary” version [27,29], which conserves the canonical ensemble, and we will also review this implementation below.

While successful in its applications, the FK-LPI model has not enjoyed as much popularity as it perhaps deserves. This may be ascribed to its rather CPU-demanding implementation: a central ingredient when calculating the Wigner-transformed Boltzmann operator is the required smeared Hessian of the potential, yielding the so-called effective frequencies. FK-LPI therefore becomes time-consuming for general potentials. However, as will be discussed below, the recent progress in neural-network-trained potentials [8,30] may potentially resolve this issue.

This review is structured as follows. In Section 2, we outline the basic equations behind the FK-LPI model and its implementation. Then in Section 3, we summarize the various applications of the method and discuss its accuracy and limitations, particularly in comparison to RPMD and CMD. Finally, in Section 4, we discuss the models with emphasis on future developments of the FK-LPI method.

## 2. The Classical Wigner Approach

Correlation functions (CFs) play an important role in the statistical mechanics of complex systems since they contain key information on dynamics. Thus, diffusion coefficients, time-dependent structure factors, infrared spectra (all exemplified below), chemical rate constants [31], and heat conductivities [32] are examples of important descriptors of chemical systems that are directly obtained from CFs. The formal expression for a quantum CF between operators $\hat{A}$ and $\hat{B}$ in the canonical ensemble is [33]

(1) $$\langle \hat{A}\left(0\right)\hat{B}\left(t\right)\rangle =\frac{1}{Z}Tr\left\{\exp\left(-\beta \hat{H}\right)\hat{A}\hat{B}\left(t\right)\right\},$$

where $\hat{B}\left(t\right)=\exp\left(-i\hat{H}t/\hbar \right)\hat{B}\exp\left(i\hat{H}t/\hbar \right)$. For instance, to obtain an infrared spectrum of a liquid, one sets both $\hat{A}$ and $\hat{B}$ equal to the total dipole moment operator of the system; see, e.g., Refs. [34,35]. There exists an alternative CF called the Kubo transform of $\langle \hat{A}\left(0\right)\hat{B}\left(t\right)\rangle$. It is defined via a thermal smearing:

(2) $${\langle \hat{A}\left(0\right)\hat{B}\left(t\right)\rangle }_{K}=\frac{1}{Z}\frac{1}{\beta \hbar }\int_{0}^{\beta \hbar }d\lambda Tr\left\{\exp\left(-\left(\beta -\lambda \right)\hat{H}\right)\hat{A}\exp\left(-\lambda \hat{H}\right)\hat{B}\left(t\right)\right\}.$$

The two CFs presented above contain the same information, since a relation in Fourier space maps one into the other [33]. Unfortunately, neither of the two CFs can be calculated for a many-body system by using the formal expressions above, as there will be a sign problem associated with the oscillatory phases of the matrix elements of the real time propagator $\exp(-i\hat{H}t/\hbar )$ [18]. The expressions have to be recast into approximate forms.

The classical Wigner model and its associated FK-LPI implementation represent perhaps the conceptually simplest approach to the calculation of general, non-linear correlation functions, which include quantum effects [14,36]. This model can be derived by *linearizing* the exact path integral representation of Equation (1) or Equation (2); see Refs. [14,15].

More precisely, the linearization of the path integral (LPI) entails modifying the action difference found in the path integral by writing $V\left(y\right)-V\left(x\right)\approx V^{\prime }\left(\left(x+y\right)/2\right)\left(y-x\right)$. If the two paths *x* and *y* are close, which they are at high temperature, this approximation will be exact. This is also the case for harmonic potentials. After the linearization, the path integral is replaced by a swarm of classical trajectories; hence, the LPI and CW methods are equivalent. The above linearization is a dynamical approximation that neglects quantum dynamical effects such as tunneling and interference. It leads to an artificial dephasing of the calculated correlation functions when studying anharmonic systems (see below). However, if the system itself has a fast natural dephasing time, as in condensed phase systems, then the CW approximation may still work well [36]. The precise expression that is derived from the linearization approximation seems to have appeared first in the work of Hernandez and Voth [37]. Although it is not a precise statement, the LPI method can be thought of as corresponding to the calculation of statistical dynamical properties by averaging over classical trajectories with initial conditions consistent with quantum mechanical position and momentum distributions (the Wigner phase space distribution). In its formal representation, it can be summarized as follows.

To obtain the correlation function $\langle \hat{A}\left(0\right)\hat{B}\left(t\right)\rangle$, one makes use of the approximate classical Wigner expression:

(3) $$\langle \hat{A}\left(0\right)\hat{B}\left(t\right)\rangle \approx$$

$$\frac{1}{{\left(2\pi \hbar \right)}^{3N}}\int \int \frac{dqdp}{Z}{\left(\exp\left(-\beta \hat{H}\right)\hat{A}\right)}_{W}\left[q,p\right]{\left(\hat{B}\right)}_{W}\left[q_{t},p_{t}\right],$$

where 3N is the dimensionality of the problem. The interpretation/implementation of Equation (3) goes as follows: phase-space points (q,p) are sampled from the Wigner transform of $\exp\left(-\beta \hat{H}\right)\hat{A}$, the transform being defined for an arbitrary operator $\hat{C}$ by:

(4) $${\left(\hat{C}\right)}_{W}\left[x,p\right]\equiv$$

$$\int_{-\infty }^{+\infty }d\eta \exp\left(-ip\eta /\hbar \right)\langle x+\frac{1}{2}\eta \left|\hat{C}\right|x-\frac{1}{2}\eta \rangle .$$

In Equation (3), (q,p) are evolved classically to $\left(q_{t},p_{t}\right)$, which serve as the phase-space arguments of ${\left(\hat{B}\right)}_{W}\left[q_{t},p_{t}\right]$.

Due to the difficulty of obtaining the exact Wigner transform of an operator, we will describe how an approximate, semi-analytic form, can be obtained. A focus will be on the Wigner transform of the Boltzmann operator, which is needed for the correlation functions that we are interested in. This calculation is particularly challenging since the Boltzmann operator contains both position and momentum operators that do not commute.

### 2.1. Feynman–Kleinert Wigner Transform

In 2003, we suggested a route to the Wigner transform of the $\exp\left(-\beta \hat{H}\right)\hat{A}$ operator, which is required for the CW model [14]. This approach is based on combining the novel effective frequency variational theory independently derived by Feynman and Kleinert (FK) [38] and Giachetti and Tognetti [39] with the quasi-density operator formalism of Jang and Voth [11]. The resulting correlation function approach, called FK-LPI, can be conceptually understood as follows. The sampling of the quantum distribution is divided into two stages. In the first stage, phase-space points are chosen based on a classical-like probability distribution which is computationally accessible. These initial points are the so-called centroids. In the second stage, a quantum distribution is sampled *around* each centroid, representing the quantum fluctuations around this classical-like point. This procedure for generating a quantum distribution can be formulated exactly. However, to make evaluation of this distribution of fluctuations analytically accessible, and therefore make the CF calculation feasible, we use a local harmonic description for the potential in the vicinity of the centroid. Further, the effective harmonic frequency description introduced by FK is used.

The effective frequencies of the local normal modes are chosen variationally, thereby optimizing the prediction of the system free energy compared to the exact result. The local harmonic description is not the same as that given simply by the curvature of the local potential, but rather accounts for the part of the potential actually sampled at the thermodynamic state. We have shown in model studies [14] that this difference is, in fact, important to the accuracy of the approach.

Presented in formal terms, in one dimension, one may approximate the Boltzmann operator by

(5) $$\exp\left(-\beta \hat{H}\right)≃\int \int dx_{c}dp_{c}\rho_{FK}\left(x_{c},p_{c}\right){\hat{\delta }}_{FK}\left(x_{c},p_{c}\right),$$

where $\rho_{FK}\left(x_{c},p_{c}\right)$ is the FK approximation to the centroid density:

(6) $$\rho_{FK}\left(x_{c},p_{c}\right)=\frac{1}{2\pi \hbar }\exp\left(-\beta \frac{p_{c}^{2}}{M}\right)\exp\left(-\beta W_{1}\left(x_{c}\right)\right),$$

and $W_{1}\left(x_{c}\right)$ is the corresponding FK approximation to the exact centroid potential $W(x_{c})$. The operator ${\hat{\delta }}_{FK}\left(x_{c},p_{c}\right)$ is the effective frequency quasi-density operator (QDO):

$${\hat{\delta }}_{FK}\left(x_{c},p_{c}\right)=\int \int dxdx^{\prime }\sqrt{\frac{M\Omega (x_{c})}{\pi \hbar \alpha }}|x^{\prime }\rangle \langle x|$$

(7) $$\times \exp\left\{i\frac{p_{c}}{\hbar }\left(x^{\prime }-x\right)-\frac{M\Omega (x_{c})}{\hbar \alpha }{\left(\frac{x+x^{\prime }}{2}-x_{c}\right)}^{2}\right\}$$

$$\times \exp\left\{-\frac{M\Omega (x_{c})\alpha }{4\hbar }{\left(x^{\prime }-x\right)}^{2}\right\}$$

where α is a function of the *effective* frequency, $\Omega (x_{c})$, through the relation

(8) $$\alpha =\coth\left(\frac{\Omega (x_{c})\hbar \beta }{2}\right)-\frac{2}{\Omega (x_{c})\hbar \beta }.$$

Wigner-transforming Equation (5) amounts to transforming ${\hat{\delta }}_{FK}$, as in Equation (7). The power of the FK approach is that this can be performed analytically:

(9) $${\left({\hat{\delta }}_{FK}\left(x_{c},p_{c}\right)\right)}_{W}\left[q,p\right]=$$

$$\frac{2}{\alpha }\exp\left(-\frac{M\Omega (x_{c})}{\hbar \alpha }{\left(q-x_{c}\right)}^{2}-\frac{1}{M\Omega (x_{c})\alpha \hbar }{\left(p-p_{c}\right)}^{2}\right).$$

Further, if $\hat{A}$ is a relatively simple operator, the transform of $\exp\left(-\beta \hat{H}\right)\hat{A}$ can also be obtained. For details, we refer to Ref. [14].

The practical implementation of FK-LPI may now be stated.

- Perform a Monte-Carlo walk in coordinates $\left(x_{c},p_{c}\right)$ using $\rho_{FK}\left(x_{c},p_{c}\right)$ in Equation (6) as a weight function.
- Sample phase-space points (q,p) around $\left(x_{c},p_{c}\right)$ as starting points for MD using Equation (9).
- The correlation function is then evaluated from Equation (3).

For a barrier, $\Omega^{2}\left(x_{c}\right)$ becomes negative and we see from Equation (9) that the momentum sampling becomes ill-defined since $M\Omega (x_{c})\alpha \hbar <0$. However, if one eliminates $p_{c}$ from the sampling function by integrating it out from Equation (5), a Gaussian sampling function depending on *p* alone remains, which is well-defined as long as $\hbar \left|\Omega (x_{c})\right|\beta <\pi$.

We should mention that other ways to obtain the Wigner transform of the Boltzmann operator exist and which therefore lead to other implementations of the classical Wigner model. For example, one may rely on a local harmonic expansion on the bare potential [36,40] or a more sophisticated thermal Gaussian approximation involving a propagation in imaginary time [41,42]. We also refer to the review of Liu [17] where some of these implementations are discussed. Central to many of these methods is the need to obtain some kind of local frequency of the system in order to evaluate the Wigner transform. We will not include a discussion of the weaknesses and strengths of other classical Wigner implementations here, since this would require a much lengthier review. However, we will point out that almost all these implementations run into trouble when dealing with systems having potential barriers. The sampling of phase-space points then breaks down at low temperature and it is not clear how to proceed. Liu and Miller [43] have however presented an elegant extrapolation procedure to imaginary frequencies that avoids this problem and this particular implementation of the Boltzmann–Wigner distribution has been shown to work well for reaction rate problems.

### 2.2. Implementing the FK-LPI Model

We will here discuss how to obtain the effective frequency $\Omega (x_{c})$ and centroid potential $W_{1}\left(x_{c}\right)$ needed for FK-LPI. For simplicity, we consider a one-dimensional particle with mass *M* and refer the reader to Ref. [23] for the multi-dimensional case. As is discussed elsewhere [23,44], the way to determine $\Omega (x_{c})$ is to iterate the two equations

(10) $$a_{FK}^{2}\left(x_{c}\right)=\frac{1}{M\Omega^{2}\left(x_{c}\right)\beta }\left\{\frac{\hbar \Omega (x_{c})\beta }{2}\coth\left(\frac{\hbar \Omega (x_{c})\beta }{2}\right)-1\right\},$$

and

(11) $$\Omega^{2}\left(x_{c}\right)=\frac{1}{M}\frac{1}{\sqrt{2\pi a_{FK}^{2}\left(x_{c}\right)}}\int_{-\infty }^{\infty }dyV^{\prime \prime }\left(x_{c}+y\right)\exp\left(-\frac{1}{2}y^{2}/a_{FK}^{2}\left(x_{c}\right)\right).$$

The smearing width $a_{FK}^{2}\left(x_{c}\right)$ has a unit of length squared and shrinks for large *T* as $\frac{\hbar^{2}}{12Mk_{B}T}$. It thus goes towards zero at high *T* while being equal to the width of the ground state wavefunction at low temperatures. Once a value of $a_{FK}^{2}\left(x_{c}\right)$ has been obtained, the smeared frequency is calculated from Equation (11). Since the smearing width itself is a function of $\Omega (x_{c})$, it follows that the two equations must be solved iteratively. Typically 5–10 iterations suffice. Once $a_{FK}^{2}\left(x_{c}\right)$ and $\Omega (x_{c})$ are known, the centroid potential is calculated according to [23]

(12) $$W_{1}\left(x_{c}\right)=k_{B}T\ln\frac{\sinh(\frac{\hbar \Omega (x_{c})\beta }{2})}{\frac{\hbar \Omega (x_{c})\beta }{2}}+V_{a_{FK}^{2}\left(x_{c}\right)}\left(x_{c}\right)-\frac{1}{2}M\Omega^{2}\left(x_{c}\right)a_{FK}^{2}\left(x_{c}\right),$$

where the smeared potential $V_{a_{FK}^{2}\left(x_{c}\right)}\left(x_{c}\right)$ is calculated as in Equation (11), but *V* replaces $V^{\prime \prime }$.

The computational bottleneck in the FK-LPI theory is the calculation of the variational effective frequency $\Omega^{2}\left(x_{c}\right)$. Only in very special cases can the smearing in Equation (11) be conducted analytically. This requires representing the atom–atom interaction potential in a special form; see, e.g., Ref. [21]. For realistic potentials, Equation (11) must instead be evaluated numerically, which is time-consuming. However, one can perform an integration by parts in Equation (11) to arrive at an equation which requires only simple force calls and a Gaussian sampling to evaluate:

(13) $$\Omega^{2}\left(x_{c}\right)=\frac{1}{M}\frac{1}{\sqrt{2\pi a_{FK}^{2}\left(x_{c}\right)}}\frac{1}{a_{FK}^{2}\left(x_{c}\right)}\int_{-\infty }^{\infty }dyV^{\prime }\left(x_{c}+y\right)y\exp\left(-\frac{1}{2}y^{2}/a_{FK}^{2}\left(x_{c}\right)\right).$$

This equation has been successfully used for obtaining the FK-LPI Wigner distribution for a graphite surface [45] composed of 300 atoms and a low-temperature helium liquid [23]. Equation (13) is important since it allows for implementing FK-LPI in a “black-box” fashion where one just needs to call an external force routine to obtain $\Omega (x_{c})$. This implementation of FK-LPI is refered to as “gradient sampling”. As an example, for a helium liquid modeled by 57 atoms in a box with periodic boundary conditions, 2500 samplings of *y* in Equation (13) were sufficient [23]. As discussed in Ref. [23], for this particular system, performing 135,000 centroid samplings through gradient sampling of Equation (13) took roughly a factor of 30/7 longer than performing 300,000 samplings using an analytical expression for Equation (11). Thus the gradient sampling approach is here only about an order of magnitude slower than using an analytically smeared Hessian.

### 2.3. The Planetary FK-LPI Model

The fundamental flaw of the classical Wigner model is clearly that the ensemble is not conserved during classical time evolution. As we have noted already, this may lead to artifacts such as zero-point energy leakage. In Ref. [29] we proposed a modification of the classical Wigner model that solves this issue.

From Equation (5), it is found that the FK-LPI model predicts the following time-dependent Wigner distribution of the Boltzmann operator:

(14) $${\left(\exp\left(-\beta \hat{H}\right)\right)}_{W}\left[q_{t},p_{t}\right]=\int \int dx_{c}dp_{c}\rho_{FK}\left(x_{c},p_{c}\right){\left({\hat{\delta }}_{FK}\left(x_{c},p_{c}\right)\right)}_{W}\left[q_{t},p_{t}\right],$$

where $\left(q_{t},p_{t}\right)$ is a classical trajectory. For a consistent model, Equation (14) should give a time-independent Wigner distribution. The FK-LPI time evolution of the initial QDO Wigner distribution ${\left({\hat{\delta }}_{FK}\left(x_{c}=x^{\prime },p_{c}=p^{\prime }\right)\right)}_{W}$, centered at $\left(x_{c}=x^{\prime },p_{c}=p^{\prime }\right)$, is defined by the cloud of classically moving phase-space points initially sampled from this distribution. At a later time *t*, the cloud defines a new distribution with position and momentum mean-values $\left(q^{\prime \prime },p^{\prime \prime }\right)$. However, this distribution is *not* equal to ${\left({\hat{\delta }}_{FK}\left(x_{c}=x^{\prime \prime },p_{c}=p^{\prime \prime }\right)\right)}_{W}$, i.e., the initial distribution at $\left(x_{c}=x^{\prime \prime },p_{c}=p^{\prime \prime }\right)$, which appears in Equation (14). Hence, there is no reason why the integral of the distributions on the right-hand side in Equation (14) should be conserved, as indeed it is found not to be.

To solve this problem, we need an algorithm where the QDO Wigner distributions ${\left({\hat{\delta }}_{FK}\left(x_{c},p_{c}\right)\right)}_{W}$ in Equation (14) move around in such a manner that when one QDO Wigner distribution replaces another, it has the same centroid density as the one it replaces. This is accomplished as follows. The center $\left(x_{c},p_{c}\right)$ of ${\left({\hat{\delta }}_{FK}\left(x_{c},p_{c}\right)\right)}_{W}$ is made time-dependent by defining $\left(x_{c}\left(t\right),p_{c}\left(t\right)\right)$ to move classically on the centroid potential $W_{1}\left(x_{c}\right)$. All points on such a trajectory have the same centroid energy and therefore $\rho_{FK}\left(x_{c}\left(t\right),p_{c}\left(t\right)\right)$ is constant along it. This prescription alone would solve the ensemble conservation problem, but we still need a way to prescribe trajectories $\left(q_{t},p_{t}\right)$ as needed in the classical Wigner model.

We next propose to let the points $\left(q_{t},p_{t}\right)$ evolve harmonically *around* the centroid $\left(x_{c}\left(t\right),p_{c}\left(t\right)\right)$, using the effective frequency $\Omega (x_{c})$. This is achieved using relative *dimensionless* coordinates:

(15) $${\tilde{q}}_{c}\left(t\right)=\sqrt{\frac{M\Omega (x_{c}\left(t\right))}{\hbar \alpha_{t}}}\left(q_{t}-x_{c}\left(t\right)\right)\;\;{\tilde{p}}_{c}\left(t\right)=\frac{1}{\hbar \alpha_{t}M\Omega \left(x_{c}\left(t\right)\right)}\left(p_{t}-p_{c}\left(t\right)\right),$$

with dynamics

(16) $${\dot{\tilde{q}}}_{c}=\Omega \left(x_{c}\left(t\right)\right){\tilde{p}}_{c}\;\;{\dot{\tilde{p}}}_{c}=-\Omega \left(x_{c}\left(t\right)\right){\tilde{q}}_{c}.$$

**Figure 1:**
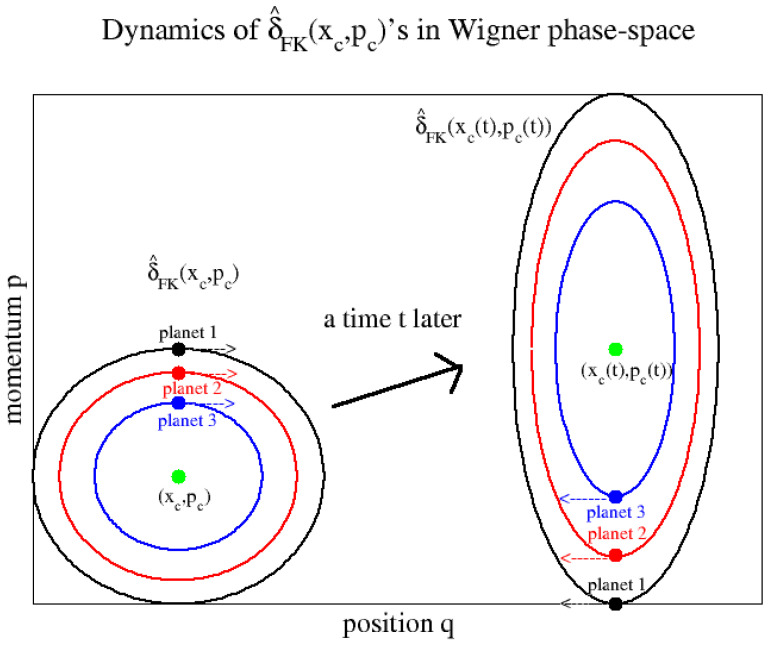
The planetary model of FK-LPI. The physical phase-space points (q,p) orbit the centroid $\left(x_{c},p_{c}\right)$ as planets around the sun.

Once the values of $\left({\tilde{q}}_{c}\left(t\right),{\tilde{p}}_{c}\left(t\right)\right)$ are known, they are easily converted back to “physical” coordinates $\left(q_{t},p_{t}\right)$ using Equation (15). The reason why we need dimensionless coordinates is that the width of the function ${\left({\hat{\delta }}_{FK}\left(x_{c}\left(t\right),p_{c}\left(t\right)\right)\right)}_{W}\left[q,p\right]$ expressed in these coordinates is always constant and will therefore be correct. When transforming back to “physical” coordinates $\left(q_{t},p_{t}\right)$ using Equation (15), the distribution will then have the correct shape, i.e., the same shape as the QDO Wigner distribution that initially was centered at $\left(x_{c}\left(t\right),p_{c}\left(t\right)\right)$. This is illustrated in Figure 1, where the shape of the QDO distribution ${\left({\hat{\delta }}_{FK}\left(x_{c}\left(t\right),p_{c}\left(t\right)\right)\right)}_{W}\left[q,p\right]$ may change which is captured perfectly by the planetary phase-space points $\left(q_{t},p_{t}\right)$ that orbit around $\left(x_{c}\left(t\right),p_{c}\left(t\right)\right)$. The algorithm then works as follows.

- Sample $\left(x_{c},p_{c}\right)$ from the Boltzmann distribution in Equation (6) (thereby giving them a thermal distribution).
- Sample phase-space points (q,p) around $\left(x_{c},p_{c}\right)$ by using Equation (9) and convert these to dimensionless coordinates $\left({\tilde{q}}_{c},{\tilde{p}}_{c}\right)$.
- Then, obtain $\left(x_{c}\left(t\right),p_{c}\left(t\right)\right)$ by performing ordinary classical dynamics on the potential $W_{1}\left(x_{c}\right)$, while simultaneously obtaining $\left({\tilde{q}}_{c}\left(t\right),{\tilde{p}}_{c}\left(t\right)\right)$ from Equation (16). Once physical particle positions or momenta are required, obtain $\left(q_{t},p_{t}\right)$ using Equation (15).

The planetary scheme is exact for harmonic potentials and at high temperatures. It has much in common with CMD, since the centers of the QDOs move precisely as in CMD, but in the planetary model there are also the physical coordinates which orbit around it. Diffusion coefficients from the planetary model therefore match those of CMD if $W_{1}\left(x_{c}\right)$ is close to the exact centroid potential $W(x_{c})$.

Two versions of the planetary model were presented in Ref. [29]. There, the planetary model described above is referred to as planetary FK-LPI(1). We will not present FK-LPI(2) here but we simply mention that the two models only differ by minor technical details.

## 3. Applications of the FK-LPI Model

The FK-LPI implementation of the classical Wigner model has been applied to many complex systems, mainly liquids [19,21,22], but also collisions between water molecules and a graphite surface have been considered [45]. Below, we will focus on the liquid studies where various correlation functions were calculated. We place emphasis on systems where we can compare our results with experimental data. Before considering realistic systems, we will study a simple model system.

### 3.1. Model Problem

**Figure 2:**
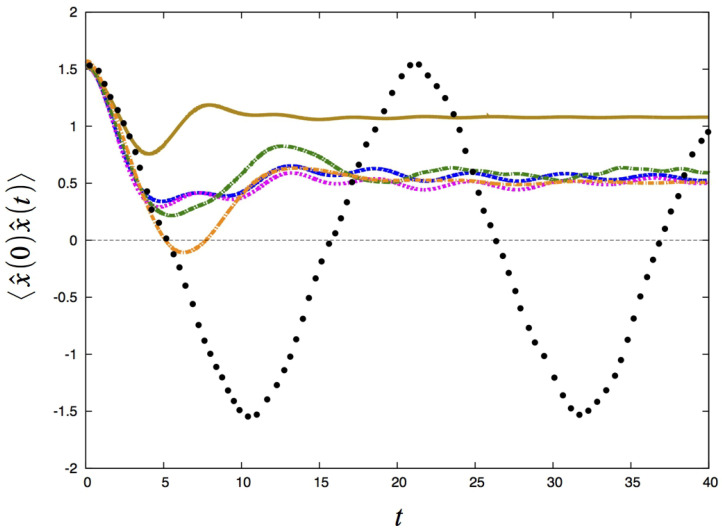
The real part of the autocorrelation function for the double well potential for a low temperature (β=8). Black points: exact; green dot-dashed line: RPMD; orange dot-dashed line: FK-CMD; gold line: FK-LPI; blue dashed line: FK-LPI(1); magenta dashed line: FK-LPI(2). Reprinted from [29].

Traditionally, new methods for calculating time correlation functions are always tested against accurate results for challenging one-dimensional model problems. The RPMD, CMD and classical Wigner methods are no exceptions; see, e.g., Refs. [10,13,29]. Here, we consider the one-dimensional model problem found in Ref. [29], where one calculates the position correlation function for a particle moving in a double well potential. For the details of the problem, we refer to [29]. We consider a temperature so low that almost only the ground state wavefunction is occupied. In Figure 2, we show calculations of $\langle \hat{x}\left(0\right)\hat{x}\left(t\right)\rangle$, defined as in Equation (1). Several approximate methods have been utilized: RPMD; FK-CMD, which is CMD using the FK approximate centroid potential; both planetary versions, FK-LPI(1) and FK-LPI(2); ordinary FK-LPI; and finally an accurate method. From the figure it can clearly be seen that the planetary versions perform essentially similar to each other and that they outperform the ordinary FK-LPI model.

### 3.2. Structure and Dynamics of Liquid Water

Water is without doubt the most important medium in which chemical and biological processes take place. As discussed below, it also exhibits certain non-classical features which make it even more interesting. CMD, RPMD and FK-LPI have therefore all been applied for studying the structure and dynamics of liquid water. We now summarize the application of FK-LPI to model room-temperature liquid water, including quantum effects.

**Figure 3:**
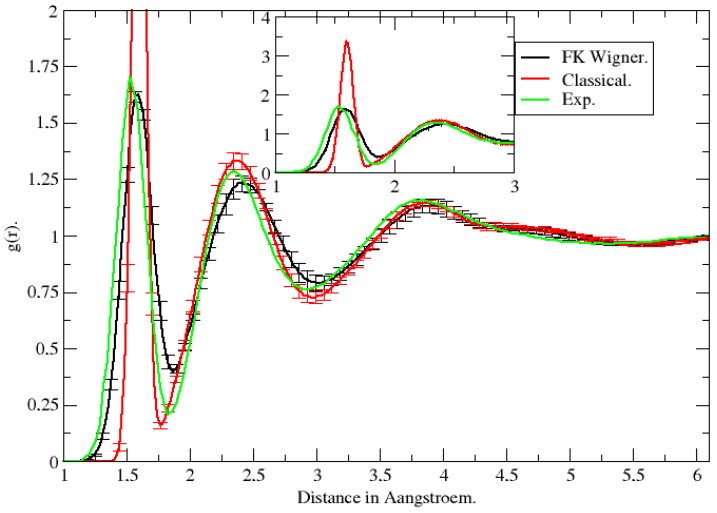
Hydrogen–hydrogen radial distribution function obtained experimentally (Exp, Ref. [48]), from classical MD (Classical, Ref. [21]) and from FK-LPI (FK Wigner, Ref. [21]).

In Ref. [21], we first considered a small box of 32 molecules which was then extended to double size in Ref. [23]. The underlying water potential model was a flexible simple point charge (SPC) model obtained by combining the rigid water SPC model of Berendsen et al. [46] with the harmonic part of the local mode intramolecular water potential of Reimers and Watts [47]. The Wigner transform was obtained for a cubic box of water molecules with periodic boundary conditions and Ewald summation for the long-range electrostatic interactions. In Figure 3 we show the hydrogen–hydrogen radial distribution function. It is clearly seen that adopting the Wigner distribution of the Boltzmann operator results in a computed radial hydrogen–hydrogen distribution function $g_{HH}\left(r\right)$ in good agreement with the experimental function [48], while a classical calculation does not.

**Figure 4:**
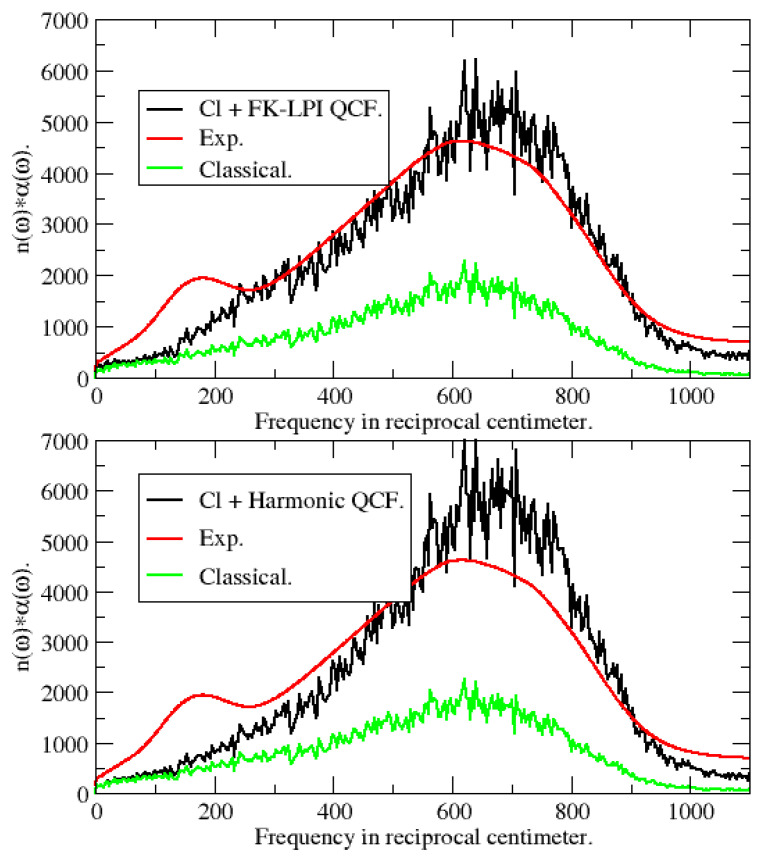
IR spectra for room-temperature liquid water obtained by applying an FK-LPI QCF (top panel) and a harmonic QCF (bottom panel) to the classical spectrum reported by Jeon et al. [49]. Cl+FK-LPI QCF = classical MD-derived IR spectrum multiplied by FK-LPI QCF. Exp. = Experimental taken from [49]. Classical = IR spectrum from classical MD. Cl+Harmonic QCF: see text. Reprinted (adapted) with permission from [23]. Copyright 2006 American Chemical Society.

In Refs. [21,23] we also computed a quantum-corrected infrared spectrum of water based on an FK-LPI derived frequency-dependent quantum correction factor (QCF), Q(ω). In short, for a given water force-field, we define Q(ω) as the ratio between the accurately calculated quantum mechanical infrared spectrum divided by its counterpart based on classical MD. As argued in Ref. [21], a dominant component in the total dipole moment CF is the hydrogen velocity correlation function (HVCF). Therefore, one may approximate Q(ω) as the ratio between the FK-LPI and classical HVCF spectra. More specifically, we apply Q(ω), obtained in this way, to the classically calculated infrared spectrum reported by Jeon et al. [49] who considered a common variant of the flexible SPC model put forth by Toukan and Rahman [50]. In Figure 4, top panel, we show the experimental IR spectrum, as well as those obtained from the total water dipole moment correlation function from MD simulations with and without Q(ω). It is clearly seen that FK-LPI is superior to pure classical MD. In the bottom of the figure, we show similar results but this time with Q(ω) replaced by a quantum correction $Q_{H}\left(\omega \right)$ based on a harmonic oscillator model [23]:

(17) $$Q_{H}\left(\omega \right)=\beta \hbar \omega /\left(1-\exp\left(-\beta \hbar \omega \right)\right).$$

It is seen that our Q(ω) performs better than $Q_{H}\left(\omega \right)$. The shoulder at ∼180 cm^−1^ seen in the experimental spectrum is absent in our theoretical spectrum. Jeon et al. have shown that reproducing this feature requires a polarizable water force-field [49].

One may also calculate the infrared spectrum of liquid water by RPMD [35,51]. As is well-known [51], the high-frequency part of the spectrum derived by RPMD is contaminated by interactions with the internal vibrational modes of the ring polymer. CMD is free of this problem. Liu and Miller [52] compared the infrared spectrum of water obtained by CMD and CW with the experimental spectrum up to 4000 cm^−1^. They found that CW overall gives a slightly better agreement with the experimental spectrum.

We also computed the water center of mass velocity autocorrelation function and obtained the quantum-corrected diffusion constant of water at 298 K. This diffusion coefficient has also been computed by RPMD [53] and CMD [54], using a rigid and a flexible SPC model, respectively. The results obtained are shown in Table 1. The quantum mechanical values are seen to be larger than those obtained from classical MD. This may be explained by the larger center of mass velocities of water molecules found in the quantum simulations. It is evident that the FK-LPI method predicts an enhancement of nearly a factor of 2.0 while the CMD and RPMD methods predict a factor of ∼1.5 compared to the classical results. Besides adopting different water potentials, the different methods also applied different box sizes and this is known to affect the value of the diffusion coefficient [53]. Accounting for this and the uncertainty limits of the calculations, the results of the three methods may be considered to agree. Still, the larger diffusion coefficient of FK-LPI compared to CMD and RPMD agrees well with the proposal of Habershon and Manolopoulos that this is explained by zero-point energy leakage in the classical Wigner model of water [28]. To understand this, remember that FK-LPI adopts classical trajectories with quantum initial conditions. This means that there is much kinetic energy available which is initially found in vibrational motion. This can leak out into intermolecular motion, as shown in Ref. [28]. This heats the liquid which implies increased speed of the molecules which enhances the diffusion constant. The leakage effect is absent in CMD, RPMD and the planetary versions of FK-LPI.

### 3.3. Structure and Diffusion in Low-Temperature Liquids

Low-temperature liquids represent a very challenging case for FK-LPI, CMD and RPMD since below some 20–30 K, quantum effects in radial distribution functions and transport properties are large. For simple atomic and diatomic liquids, the interaction potentials are furthermore well-known and any disagreement between simulation and experiment may be ascribed to the shortcomings of the former.

The FK-LPI method has been applied to study quantum effects in para-hydrogen and normal He at low temperatures. We first consider liquid para-hydrogen, which we studied at T=17 and 25 K at correct quantum mechanical densities [19]. The para-hydrogen molecule possesses a rotor wavefunction that is spherically symmetric for the J=0 (para) state. Thus, the interaction between two such molecules depends only on their center-of-mass distance. As a consequence, the para-hydrogen molecule may be represented simply as an atom, which we did [19].

**Figure 5:**
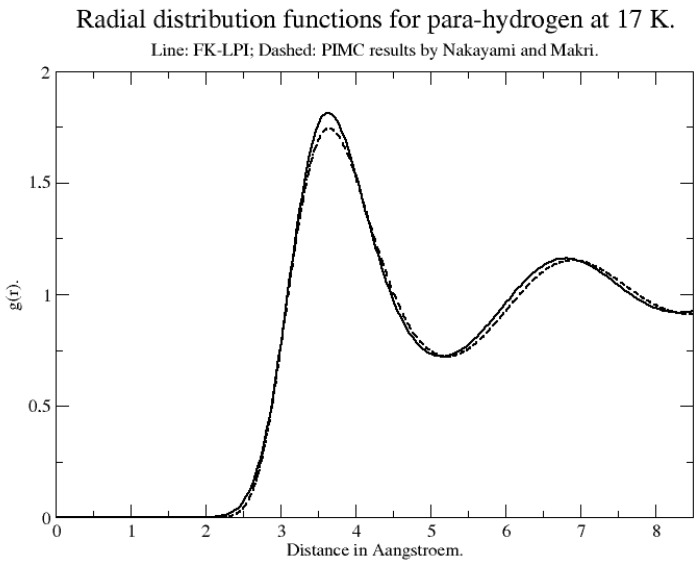
Comparison between radial distribution functions obtained from accurate path integral Monte Carlo [55] and an approximate FK Wigner distribution function. Reprinted with permission from [19]. Copyright 2004 American Chemical Society.

We start by assessing the accuracy of the FK Wigner transform by comparing its predicted para-hydrogen radial distribution function with an accurate one obtained from path integral Monte Carlo calculations as reported by Nakayama and Makri [55] using the same potential and thermodynamic state. The two functions are shown in Figure 5. We see good agreement and this shows us that the FK theory is able to model the structure of the liquid very well. We do not report any results based on classical MD because the system is not describable as a liquid at these temperatures when using correct densities but only as a classical Boltzmann weight function. In fact, as shown by Miller and coworkers [56], classically the liquid is unstable and bubbles are formed within it.

In Table 2, we show the calculated diffusion coefficients obtained from FK-LPI, CMD and RPMD when applied to para-hydrogen (we choose to report FK-LPI diffusion coefficients based on the FK-LPI Kubo CF and not the FK-LPI normal CF, since the former may be shown to predict a more consistent kinetic energy of the para-hydrogen molecules [19]). For this liquid, taking into account the challenging nature of the system, particularly at 17 K, there is good agreement between the calculated and experimental results. The problem of zero-point vibrational energy leakage alluded to above is absent for para-hydrogen. Yet, the FK-LPI diffusion coefficients are larger than those of RPMD and CMD. Still, the predictions of RPMD, CMD and FK-LPI all agree quite well with each other.

Next we consider an even more challenging problem: normal He at the very low temperature of T=4 K. In Ref. [20], CMD, FK-LPI and RPMD were all used for obtaining quantum mechanical diffusion coefficients and the results are shown in Table 3. Again, without any zero-point vibrational energy leakage, we see that the FK-LPI diffusion constant is somewhat larger than that of RPMD and CMD. However, given the strong quantum effects in this system, the agreement must be considered to be very good.

Above, we have not shown any diffusion coefficients obtained by planetary FK-LPI. However, since the FK approximation to the exact centroid potential is usually very good, we expect the result to be very close to that of CMD.

### 3.4. Dynamic Structure Factors from FK-LPI

**Figure 6:**
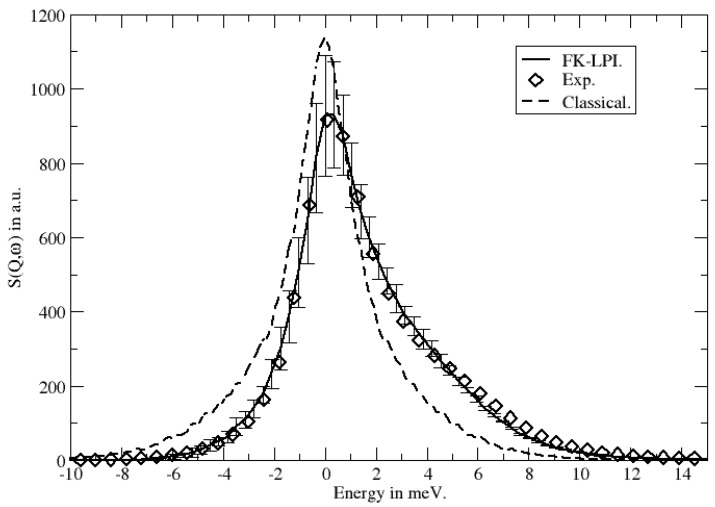
Dynamic structure factors of liquid neon as calculated by FK-LPI and classical MD. Experimental results are shown as well. Reprinted from [24].

In physics, the dynamic structure factor is a mathematical function that contains information about time-dependent density correlations with a certain periodicity, specified by a vector $\vec{Q}.$ The dynamic structure factor, or equivalently the Van Hove correlation function, is calculated as

(18) $$S\left(\vec{Q},t\right)=\frac{1}{Z}Tr\left\{\exp\left(-\beta \hat{H}\right)\sum_{j,j^{\prime }=1,N}\exp\left(-i\vec{Q}\cdot {\hat{r}}_{j}\right)\exp\left(i\vec{Q}\cdot {\hat{r}}_{j^{\prime }}\left(t\right)\right)\right\},$$

where ${\hat{r}}_{j}\left(t\right)$ is the time-dependent position Heisenberg operator of atom *j*. FK-LPI, both in its standard and planetary versions, has been applied to the calculation of dynamic structure factors of liquids on several occasions. More specifically, the following low-temperature liquids have been considered using the planetary model: a neon–deterium mixture (Colognesi et al. [25]), pure ortho-deuterium (Guarini et al. [26] and Smith et al. [27]) and para-hydrogen (Smith et al. [27]). The standard FK-LPI model has been applied to helium (Poulsen et al. [22]), pure ortho-deuterium and para-hydrogen (Smith et al. [61]) and pure neon (Scheers et al. [24]). In all of these applications, experimental dynamic structure factors have been available and the FK-LPI spectra, I(q,ω), generally agree well with these. As an example, in Figure 6 we show the dynamic structure factors calculated from standard FK-LPI and classical MD for a neon liquid at 27 K [24]. Also the experimental spectrum is shown. The quantum effects that are exhibited by this system are well represented by the FK-LPI model.

**Figure 7:**
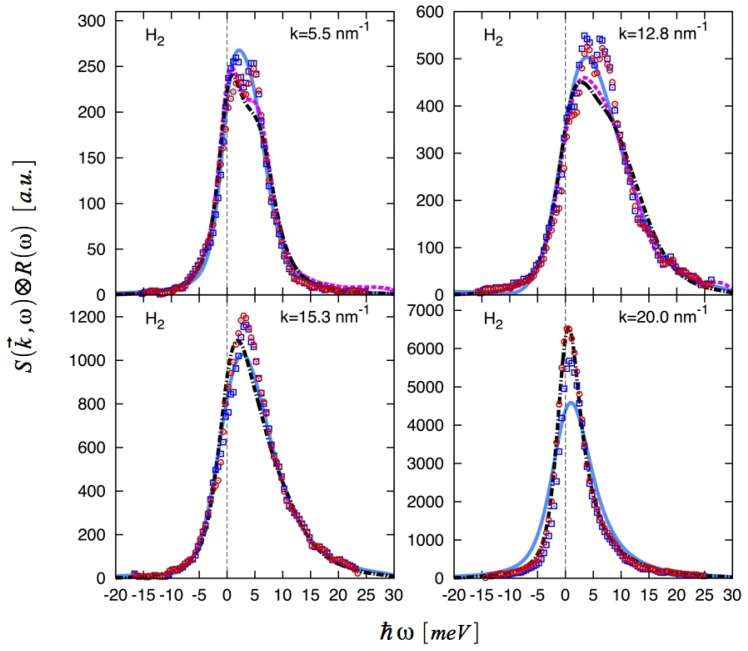
Experimental dynamic structure factors for para-hydrogen, as obtained using either FK-LPI(2) (red dots) or standard FK-LPI (blue squares) as theoretical input. The FK-LPI(2) (black dashed dot line), standard FK-LPI (blue line), and RPMD (magenta dashed line) dynamic structure factors have been convoluted with the instrumental resolution function. Reprinted from [27].

Generally speaking, the quality of dynamic structure factors derived from FK-LPI are on par with spectra calculated using RPMD; see, e.g., the work by Guarini et al. [26] and Colognesi and coworkers [25]. For large *q* vectors, where I(q,ω) is derived from a highly non-linear van Hove correlation function, FK-LPI has a clear advantage over RPMD [25]. The performance of RPMD, standard FK-LPI and planetary FK-LPI(2) can be assessed by considering Figure 7, which shows experimental and theoretical dynamic structure factors for para-hydrogen taken from Ref. [27]. In this work, an elaborate correction of the raw experimental I(q,ω) data was applied where the correction required knowledge of a theoretical spectrum of density fluctuations. In this way, the final experimental I(q,ω) depends on what theoretical model one adopts and more than one corrected experimental spectrum is therefore shown in Figure 7. Several observations can be made. We see that for q=5.5 nm^−1^ and q=12.8 nm^−1^, standard FK-LPI performs slightly better than both planetary FK-LPI(2) and RPMD. For these *q*-values, the latter two methods produce very similar spectra. At the higher *q*-values, RPMD does not produce meaningful results and is not shown. In this regime, planetary FK-LPI(2) instead outperforms standard FK-LPI. Indeed, for q=20.0 nm^−1^, the red experimental spectrum based on FK-LPI(2) matches the experimental convoluted FK-LPI(2) result (red dots).

## 4. Discussion and Outlook

We have discussed and presented results of the so-called FK-LPI implementation of the classical Wigner or linearized path integral model. It is designed to approximately include quantum effects into classical molecular dynamics by sampling initial conditions from a quantum Wigner distribution function. The method is very general and can be applied to processes taking place in solid, liquid and gas phases.

The value of FK-LPI as a tool in molecular simulations depends to a large extent on its accuracy in calculating quantum correlation functions, its ease of implementation and its CPU usage. From model problems [29], it has been shown that the ensemble-conserving planetary FK-LPI model is more accurate than the ordinary FK-LPI model, where the latter uses ordinary classical trajectories. The planetary model is observed to be of similar accuracy as the established RPMD and CMD methods (see discussion and results above). Since the planetary version is not more computationally demanding than the ordinary FK-LPI, it is clearly the most promising implementation of the two.

We next mention some chemical problems for which FK-LPI, or CW in general, is expected to be more accurate than CMD and RPMD. We thus turn to problems involving a strongly non-linear correlation function. We have already mentioned that the non-linear dipole moment correlation function was found to be more accurately calculated by the CW method than CMD [52]. Another interesting case is the calculation of the thermal conductivity of para-hydrogen at low temperature. In this case, the conductivity is given by the non-linear energy current autocorrelation function [62]. Liu and coworkers have applied the CW model to this problem for a set of low temperatures and obtained conductivities within 20 percent of the experimental values. This is in much better agreement with the experimental values than predictions from the CMD method [32]. As a final example of a problem involving an extremely non-linear correlation function, we mention the case of vibrational energy relaxation of an oxygen molecule in liquid oxygen at 70 K [40]. Here, the CW approximation to correlation functions was successfully applied for extracting the relaxation rate from the so-called force–force autocorrelation function.

We conclude by pointing to the biggest disadvantage of both the ordinary and planetary FK-LPI models and discuss a possible improvement. The biggest disadvantage of the FK-LPI method must be considered to be its intense CPU usage, since it requires the sampling of thousands of potential gradients around the centroid to obtain $\Omega (x_{c})$, via Equation (13). On top of that, one needs to repeat this procedure in each iteration step of Equations (10)–(13) (these demanding steps are required for both planetary and ordinary FK-LPI). We also observe that in the multi-dimensional case, the iterative equations Equations (10)–(13) require the diagonalization of the effective frequency matrix in order to evaluate Equation (10). In applications up to several hundreds of atoms, this diagonalization has not been observed to be the computational bottleneck, but eventually it will be since it scales as $N^{3}$, where *N* is the number of atoms. In this regard, RPMD and CMD have the advantage of better scaling.

To make FK-LPI computationally more efficient, it is vital to devise a more efficient implementation of the iterative Equations (10)–(13). Initial steps toward this goal have already been taken. In particular, Willatt et al. [63] have in their study of the Matsubara dynamics of water and ice shown that one may devise a non-iterative approximation to $\Omega (x_{c})$ involving only a first-order derivative of the potential. This is essentially accomplished by replacing the weight function in Equation (13), which depends on $\Omega (x_{c})$ (through $a_{FK}^{2}\left(x_{c}\right)$) by a new one. The new one is *independent* of $\Omega (x_{c})$, and uses a weight function derived from the exact imaginary time action. In Ref. [63] it is shown that effective frequencies derived from this non-iterative version are very close to those obtained from the original FK iterative equations. The authors conclude that this non-iterative implementation of the FK equations is “taking about the same time as a CMD calculation since it involves the sampling of the fluctuations about the centroid” [63].

While the procedure just described eliminates the costly iterative nature of the effective frequency algorithm, there remains the problem of sampling and evaluating a vast number of potential energy gradients around $x_{c}$ in order to obtain $\Omega^{2}\left(x_{c}\right)$. This procedure also remains a computational bottleneck in the implementation of Ref. [63]. It has been demonstrated that FK-LPI based on the black-box gradient sampling technique (Equation (13)) *can* quantize a graphite surface by obtaining its Wigner transform for an analytic potential [45]. However, it is still too time-consuming to sample potential energy gradients using DFT. The question is therefore as follows: can we eliminate the need for sampling thousands of potential energy gradients around the centroid? That is, can we abandon Equation (13) completely but still find a way to obtain $\Omega^{2}\left(x_{c}\right)$? We think this is possible, as explained below.

We first observe that to a very good approximation, $M\Omega^{2}\left(x_{c}\right)$ equals the Hessian of the centroid potential $W_{1}\left(x_{c}\right)$. This has been verified by studying various anharmonic potentials, including a double well. Since $W_{1}\left(x_{c}\right)$ in turn is a very good approximation to the exact centroid potential known from CMD, called $W(x_{c})$, this transforms the problem into one of computing $W(x_{c})$ and its Hessian efficiently. Recently, Loose et al. [30] presented machine-learned CMD (ML-CMD), which is an implementation based on a deep learning neural network (DeepMD) that replaces the costly CMD potential with a cheaper analytical neural network force field (NNFF). By training a DeepMD on short trajectories using full path integral MD to obtain $W(x_{c})$, they could afterwards perform much faster CMD dynamics using the fast NNFF.

Loose et al. did not need the Hessian of $W(x_{c})$ in their work as we do for FK-LPI. However, since the NNFF is analytical, we can differentiate it directly to obtain its Hessian and thereby $\Omega^{2}\left(x_{c}\right)$. Very recently, Juan et al. [64] demonstrated precisely the success of such a procedure. They trained the neural network-based potential NewtonNet against energies and forces using a large potential energy and force dataset. By efficiently differentiating NewtonNET twice, Hessians could be constructed several orders of magnitude faster than by using DFT. Clearly such a strategy seems promising and an approach for calculating Hessians using differentiable neural network based potentials will be the subject of future research.

## Figures and Tables

**Table 1:** Computed diffusion constants obtained from FK-LPI, RPMD and CMD. FK-LPI and CMD employed flexible simple point charge models of water, while that used for RPMD was rigid. The QM diffusion constants from FK-LPI are derived using either Kubo or normal velocity correlation functions, while RPMD and CMD rely on the Kubo version. All units are in ${\overset{˚}{A}}^{2}$/ps.

	FK-LPI Study [21]	RPMD Study [53]	CMD Study [54]
Cl	0.25±0.06	0.29	0.30
QM	0.47±0.06 (normal); 0.49±0.06 (Kubo)	0.43	0.42

**Table 2:** Diffusion coefficients for liquid para-hydrogen obtained from the Kubo velocity autocorrelation function. All units are in ${\overset{˚}{A}}^{2}$/ps.

	Para-Hydrogen FK-LPI Study [19]	CMD Study	RPMD	Exp. [57]
T=25 K	1.73	1.54 [58]	1.48 [59]	1.6
T=17 K	0.83	0.47 [60]		0.68

**Table 3:** Diffusion coefficients for liquid He obtained from the Kubo velocity autocorrelation function, taken from Ref. [20]. The units are in ${\overset{˚}{A}}^{2}$/ps.

He Liquid	FK-LPI	CMD	RPMD
T=4 K	0.58	0.52	0.45

## Data Availability

The raw data supporting the conclusions of this article will be made available by the authors on request.
